# Spintronic terahertz emitters with integrated metallic terahertz cavities

**DOI:** 10.1515/nanoph-2023-0807

**Published:** 2024-03-01

**Authors:** Martin Mičica, Adrien Wright, Pierre Koleják, Geoffrey Lezier, Kamil Postava, Jacques Hawecker, Anna De Vetter, Jerome Tignon, Juliette Mangeney, Henri Jaffres, Romain Lebrun, Nicolas Tiercelin, Mathias Vanwolleghem, Sukhdeep Dhillon

**Affiliations:** Laboratoire de Physique de l’Ecole Normale Supérieure, ENS, Université PSL, CNRS, Sorbonne Université, Université Paris Cité, F-75005, Paris, France; Univ. Lille, CNRS, Centrale Lille, Univ. Polytechnique Hauts-de-France, UMR 8520-IEMN, F-59000, Lille, France; IT4Innovations National Supercomputing Center & Faculty of Materials Science and Technology, VSB - Technical University of Ostrava, 17. listopadu 15, 708 00 Ostrava, Czech Republic; Femtosecond Spectroscopy Unit, Okinawa Institute of Science and Technology Graduate University, Onna-son, Okinawa, 904-0495, Japan; Laboratoire Albert Fert, CNRS, Thales, Université Paris-Saclay, 1 Avenue Augustin Fresnel, 91767, Palaiseau, France

**Keywords:** THz spintronic emitters, THz time domain spectroscopy, metallic resonator

## Abstract

Spintronic terahertz emitters (STEs), based on optical excitation of nanometer thick ferromagnetic/heavy metal (FM/HM) heterojunctions, have become important sources for the generation of terahertz (THz) pulses. However, the efficiency of the optical-to-THz conversion remains limited. Although optical techniques have been developed to enhance the optical absorption, no investigations have studied the application of THz cavities. Here, to enhance the THz efficiency of STEs in a selected THz spectral range, FM/HM structures are realized on ultra-thin sapphire layers with metallic mirrors to create *λ*/4 THz resonant cavities. THz emission time domain spectroscopy of these STE/sapphire/mirror heterostructures, with sapphire thicknesses ranging from 110 µm to 25 µm, shows enhancement of the emitted THz field that fits the *λ*/4 cavity resonance with up to a doubling of the field in the spectrum, and in agreement with temporal simulations of the emitted THz pulse. By taking advantage of birefringent materials, we further show the potential of control of the polarization state of the emitted THz pulse. This work shows the potential of enhancing and engineering THz emission from STEs using THz cavities that can be controlled over a broad spectral range, which can be easily combined with optical cavities.

## 1 Introduction

Spintronic terahertz emitters (STEs) are promising sources for the generation of THz short pulses by near-infrared (NIR) femtosecond excitation for THz time domain spectroscopy (TDS). Over a short period of time, they can now compete with well-established THz sources such as optical rectification in nonlinear crystals. There are several advantages of STEs including their very broad spectral bandwidth, negligible dependence on optical pump wavelength, manipulation of the polarization of the emitted THz pulse by an applied magnetic field, and no requirements for electric bias or crystal orientation [1], [2], [3], [4], [5]. Further, the active material is only a few nanometers thick with easily manipulated materials, making it possible to deposit these STEs on any supporting substrate or support [6], [7]. These features make STEs a versatile and novel source for generating THz radiation for a wide range of applications, bringing new device concepts that would not be possible with other types of THz sources.

The operation of an STE is based on spin-to-charge conversion (SCC) via the inverse spin-Hall effect (ISHE) [8], [9]. A typical STE consist of ferromagnetic (FM) and heavy metal (HM) layers with thicknesses on the order of a few nanometers. When excited by a NIR femtosecond pulse, electrons (majority with spin-up) in the FM layer are excited above their Fermi level and a spin polarized current flows through the spin selective interface between the FM and HM. This spin current is converted to short charge current in the HM by the ISHE and results in the emission of a THz pulse. The polarization of emitted THz pulse is perpendicular to the magnetization of FM layer and the spin current. A range of studies have investigated different metallic systems to optimize the conversion efficiency and bringing insights into the SCC processes [10], [11], [12]. Beyond the inverse SHE, which is purely a bulk effect, extensive investigations have investigated STEs through the inverse Rashba–Edelstein effect that occurs at quantum interfaces such as in FM/topological insulator heterostructures [13], [14], [15].

Photonic-based techniques have become important to increase the THz efficiency of STEs and take advantage of the integration possibilities of STEs. For example, cavities based on photonic crystals or dielectric mirrors have been investigated to maximize the NIR pump absorption in the FM layers, permitting to increase the NIR to THz conversion by up to 2.5 times [16] and permitting peak fields greater than 1.5 MV/cm [17], [18] in large area STEs pumped by amplified laser systems. However, no photonic methods have been applied directly on the THz pulse generated. In this work, we demonstrate the realization of STEs with integrated THz quarter wavelength cavities, permitting to enhance and control the THz spectral emission from these structures. We exploit metallic-based THz cavities (quasi Fabry–Perót) [19] where STEs are deposited on ultra-thin sapphire substrates with a back metal mirror. The acquired data are supported by theoretical simulations in the time and frequency domains and provides an important tool for further improvements in the performance and engineering of THz cavities. Additionally, we take this THz cavity approach and demonstrate the concept of polarization control of the THz radiation utilizing a cavity constructed around a birefringent medium.

## 2 Concept of THz cavity

**Figure 1:**
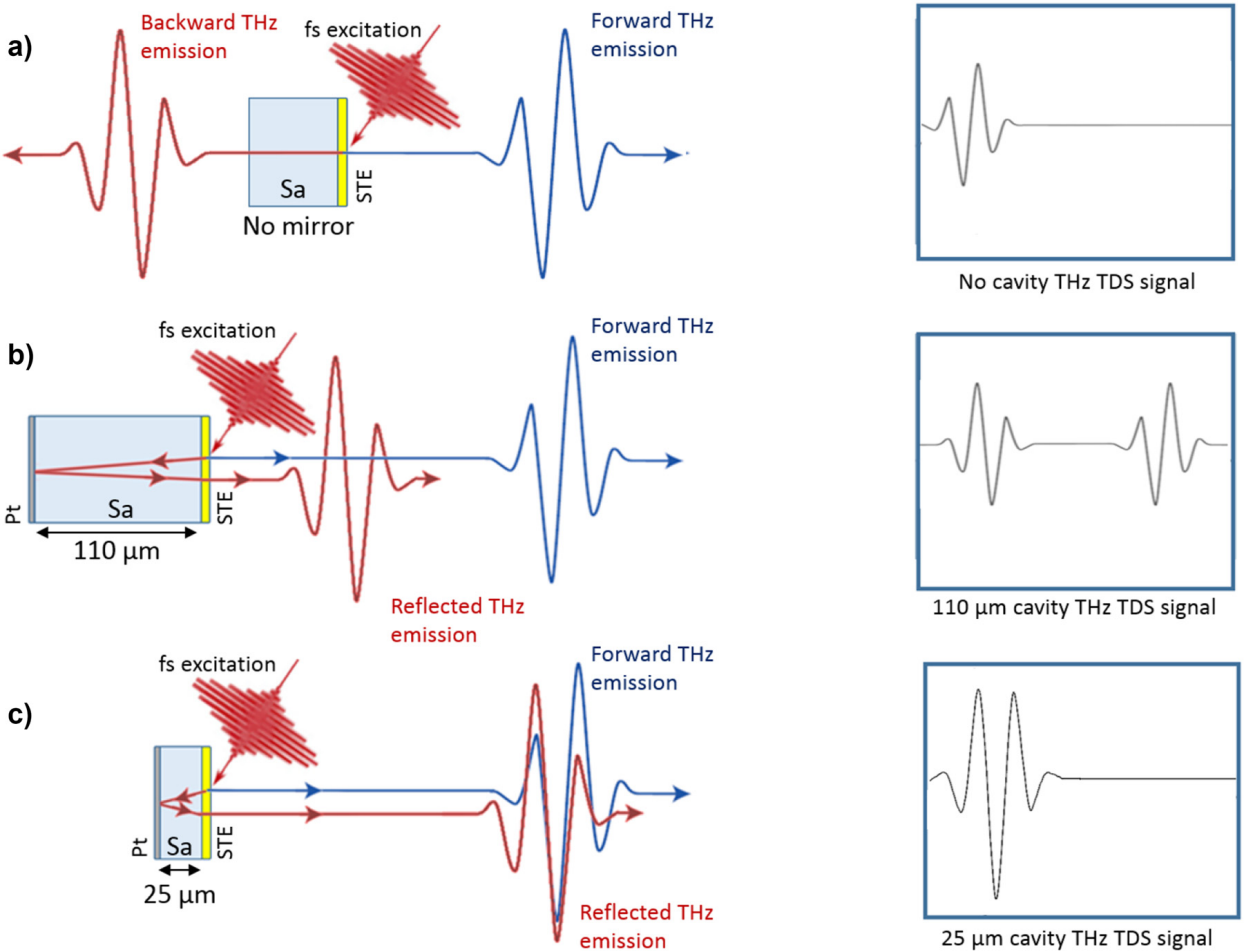
THz signal enhancement principle based on THz cavities optically excited with a femtosecond laser. (a) Standard STE emitting THz pulses in both left and right directions (reflection from substrate not shown), (b) collection of the second pulse with 110 µm thick substrate with back metal mirror, and (c) adjustment of the substrate thickness to 25 µm to realize a THz cavity and hence a single pulse for maximum THz field strength.

Figure 1 shows conceptually our approach where a THz pulse is generated in an STE using a femtosecond laser. The THz pulse generated in the FM/HM layer propagates in both left and right directions with similar amplitudes, as shown in Figure 1(a). Owing to the lower contrast of refractive indices between metal/substrate (usually glass or sapphire) compared to metal/air, more power can be emitted into the side with the substrate. In the reflection configuration, when the STE is optically pumped directly (not through the substrate) and the THz pulse is collected from the same side, the second pulse propagating via the substrate can be reflected in the same direction by incorporating a metallic mirror. This results in two THz pulses in the time domain, with a delay given by thickness of substrate and refractive index of the substrate, as depicted on Figure 1(b). The second pulse will exhibit an opposite phase owning to the reflection from the metal. Note that this can be observed only for substrates with low absorption in THz spectral range, such as sapphire or high resistivity silicon. By choosing the right thickness of the substrate, typically corresponding to a quarter wavelength condition, the delay between pulses can be decreased to the level that the pulses overlap and their electric field sum together (Figure 1(c)). Assuming that both pulses have similar amplitudes, the final amplitude can be potentially doubled or even further enhanced at resonant conditions (i.e., where thickness, *d* = *λ*/4*n* where *λ* is the wavelength and *n* is refractive index) [19].

## 3 Samples and experimental setup

To realize the THz cavity, spintronic emitters were fabricated on double side polished sapphire substrates (A cut, $1\bar{1}02$ ) with varying thicknesses ranging from 111 µm to 25 µm (chosen to highlight the effect of cavity thicknesses – see simulations below). These substrate types were also chosen to highlight the possibility of adding an extra functionality in the control of the THz polarization (see below). An optimized spintronic trilayer was deposited by magnetron sputtering on the top side consisting of W(2 nm)/CoFeB(1.8 nm)/Pt(2 nm). On the opposite side of the substrates, a thick metal layer (Pt, approx. 200 nm) was deposited, acting as metallic mirror and thus creating the THz cavity. After this growth preparation, the thicknesses of the samples were verified by a calibrated gauge that confirmed thicknesses of 111 µm, 39 µm, 29 µm, and 25 µm.

**Figure 2:**
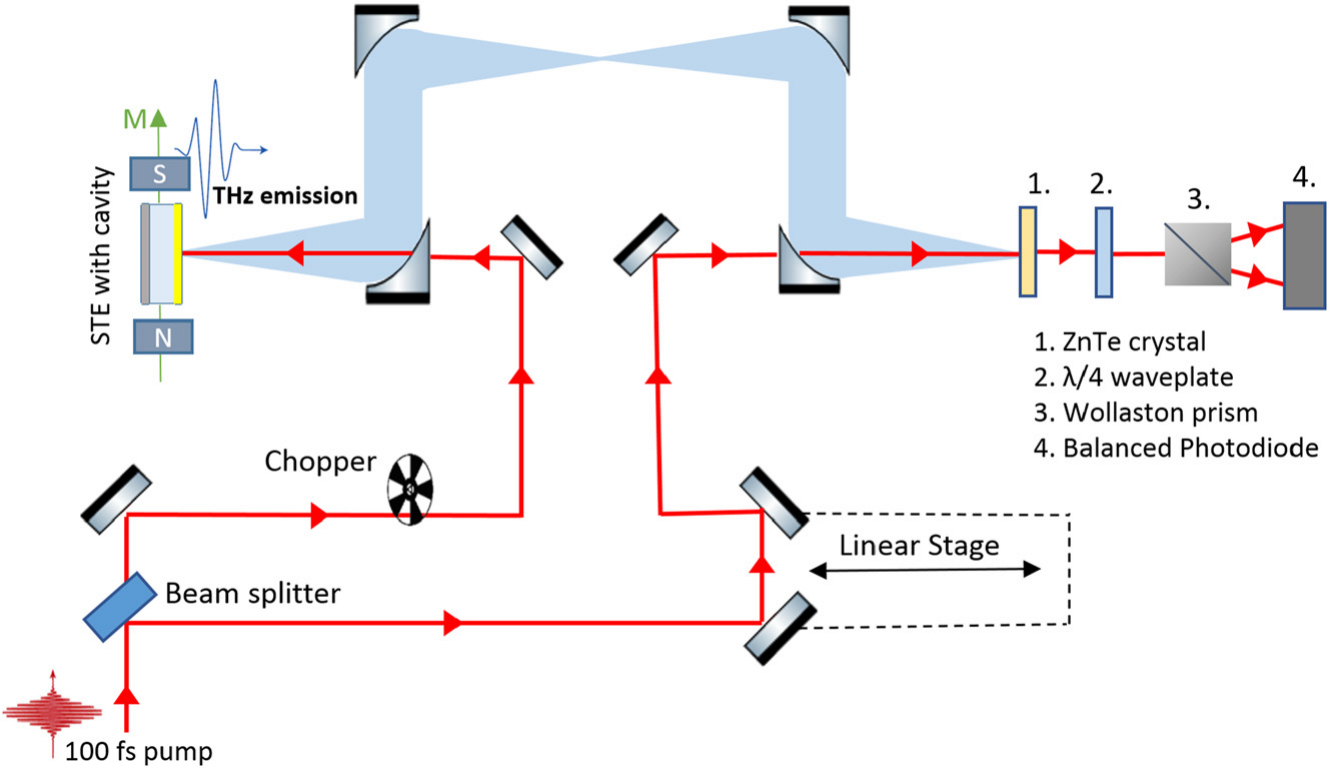
Scheme of terahertz time domain setup in reflection configuration where STE is excited with a femtosecond laser, generating a THz pulse that is detected using standard electro-optic sampling.

A custom THz TDS system, shown in Figure 2, was employed to study THz pulse generation from the prepared STEs. A NIR pulse from a Ti:Sapphire oscillator (Coherent Mira, 100 fs, central wavelength 800 nm, 200 mW average power after optical chopper) was focused by a lens through the hole in the back of a parabolic mirror onto the sample under normal incidence. The emitted THz pulses from the STEs were collected by the same parabolic mirror and focused by a set of parabolic mirrors on to an electro-optic crystal (ZnTe, 1 mm) for coherent sampling using a probe NIR beam from the oscillator. The delay of the probe was controlled by a linear motor stage. The probe was focused through the hole in the last parabolic mirror to superpose with the THz pulse on the electro-optic crystal. An electro-optic signal with sensitivity to the horizontal THz polarization with femtosecond resolution was then measured by a Wollaston prism, a balanced photodetector and the delay line. The STE samples were mounted in a rotation stage with permanent magnets (approx. 30 mT field) with independent rotation of the in-plane magnetic field and the sample. The THz beam path was purged by dry air to remove water vapor absorption. Teflon and polyethylene filters were used to remove residuals of the NIR pump pulse.

## 4 Results and discussion

**Figure 3:**
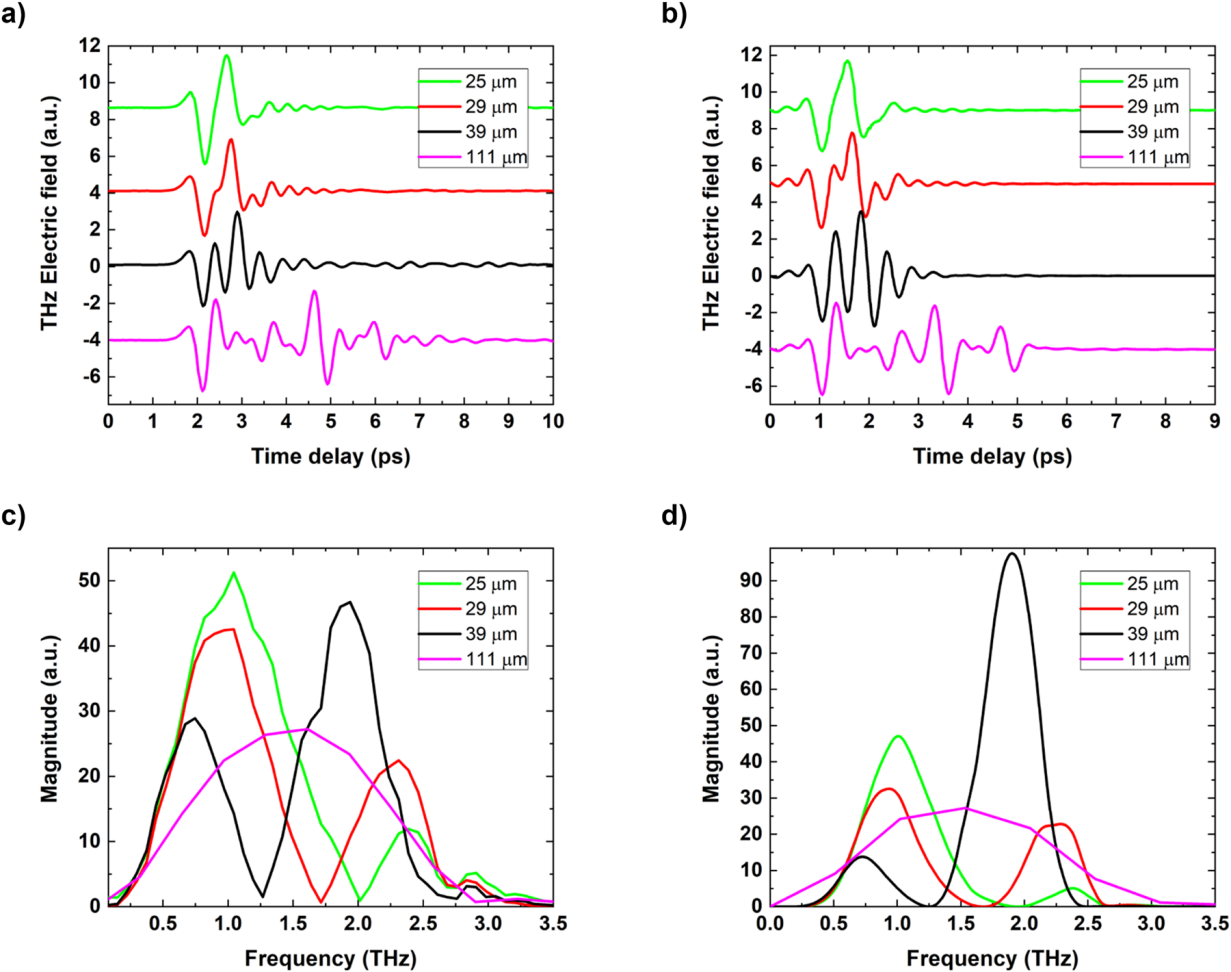
Comparison of experimental and simulated THz emission from spintronic THz cavities. (a) Experimental and (b) simulated THz TDS temporal signals, (c) experimental spectra and (d) simulated spectra of the signals for four different size sapphire cavities. Simulations of (b) and (d) were calculated by using the Scattering Matrix formalism. Spectra for the 111 µm sample is apodized only around the first pulse to remove the interference pattern.

The measured THz time domain signals of the different STEs are shown in Figure 3(a). The signal from the thickest 111 µm sample shows a temporal trace with a total of four pulses, the forward and the reflected, separated by 2.5 ps on their arrival time at the detection scheme.

The extra optical path length *p* that the THz pulse propagates can be approximated (by neglecting both interfaces Pt/sapphire and sapphire/STE) by the formula *p* = 2 × *d* × *n*_THz_, where *n* is the refractive index of the cavity medium (sapphire fast axis, 1.76 @ 800 nm [20], 3.07 @ 1 THz [21]) and *d* is thickness of the cavity. As the 111 µm cavity is thick, the overlap of the different generated THz pulses is effectively zero. The first detected pulse at ∼2 ps corresponds to the main pulse propagating toward the collecting parabolic mirror after excitation by the NIR pulse. Owing to the transparency of the sapphire substrate, the NIR pump pulse residue after excitation of the STE propagates inside the sapphire substrate and is reflected from the Pt mirror back toward the STE layer, which generates a second THz pulse at ∼3.5 ps about 0.5× of the first pulse amplitude. As the optical path length for NIR pulse is short, owing to the low refractive index, the detected THz signal is close to the first THz pulse. This is followed by the third THz pulse at ∼4.5 ps, which corresponds to the reflection of the first THz pulse propagating in opposite direction (into the substrate) and then reflected by the back Pt mirror. The fourth THz pulse at ∼6 ps is the reflection of the second NIR excitation of STE that propagates back and forth from the substrate. As the STE layer behaves as antireflection coating in THz frequencies [22], further reflections between STE and Pt mirror are not observed, and further excitations by the propagating NIR pump are negligible owing to their reduced intensities. The reflection of THz pulses from Pt mirror causes a phase reversal for the third and fourth THz pulses. The phase of the second one is not reversed as it originates from the STE excitation, which is independent of the phase and polarization of the NIR pump and is dependent only on orientation of the magnetic field.

As the thickness of the substrate is decreased, the temporal form changes drastically. As noted above, for the 111 µm sample (Figure 3(a)), we can observe and identify individual pulses. However, for the 39 µm thick cavity, the pulses start to sufficiently overlap to create a complex and long oscillating profile of the THz signal. For the 29 µm cavity, one pulse is formed with slight deformations. For a cavity thickness of 25 µm, the pulses overlap enough to create one single cycle with a slight oscillating tail after the main pulse.

The THz emission spectra of the STE samples on 111 µm, 39 µm, 29 µm, and 25 µm sapphire substrates are shown in Figure 3(b). The spectra are obtained by applying a Fast Fourier Transform on the time domain signals.

The sum of the multiple time shifted pulses in the time domain creates a magnitude modulation of the signal in the frequency domain owning to the cavity resonances. The typical reference STE spectrum, i.e., without the effect of the substrate/cavity, is obtained by apodization of the time trace of the 111 µm sample around the first pulse. The spectrum is continuous, with a 2 THz bandwidth centered at a frequency of 1.5 THz. This also highlights the performance benchmark of our THz TDS system with a detection bandwidth of up to 3.5 THz. The spectral bandwidth is dominantly limited by the length of NIR probe pulse and properties of electro-optic crystal [23], [24] (e.g., thickness).

**Figure 4:**
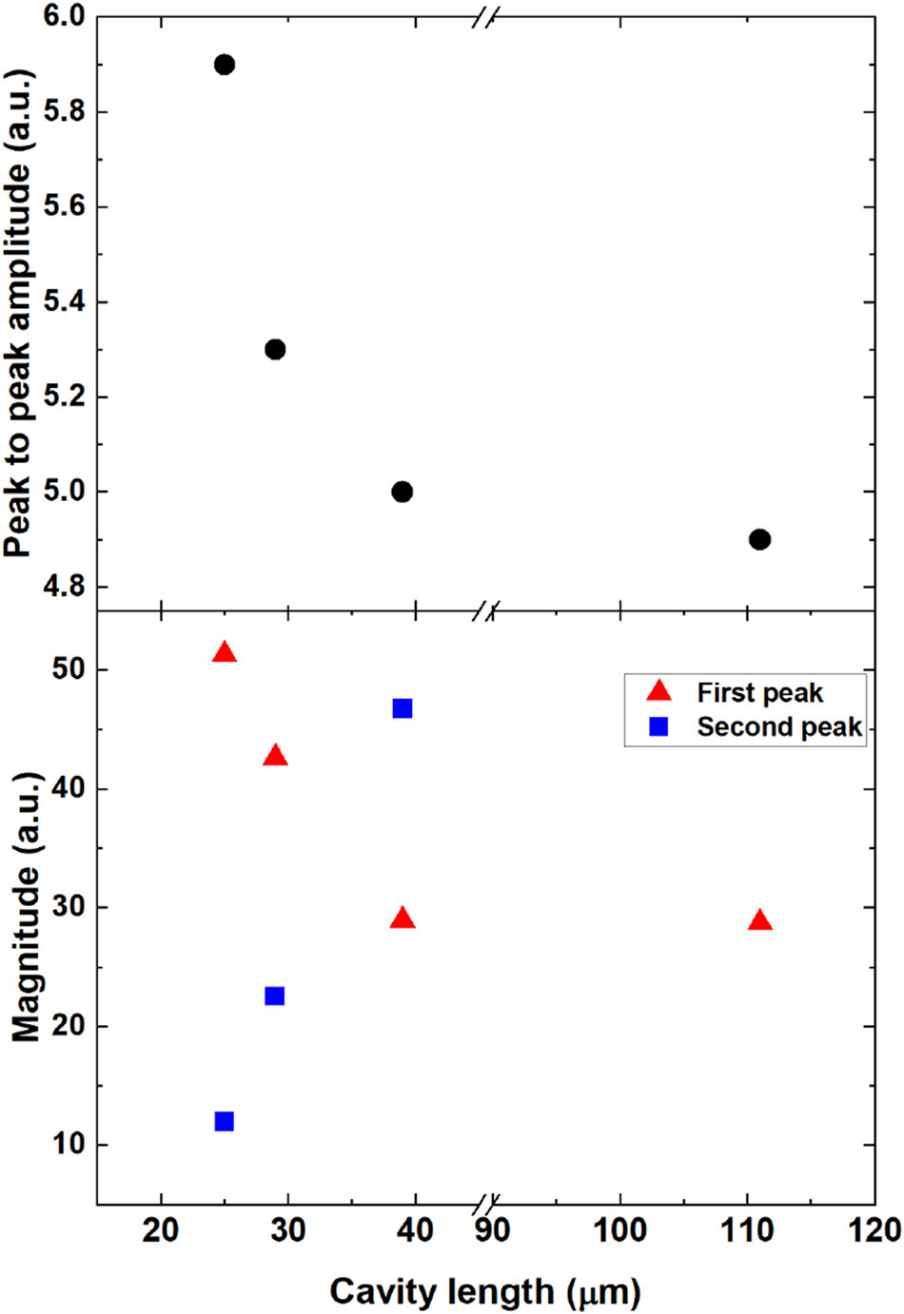
Peak-to-peak value of the temporal signals (top) and spectral magnitude of the two emission peaks (bottom) as a function of sapphire substrate thickness.

For the spectrum of the 39 µm thick cavity (without time domain apodization), we observe an interference pattern with two maxima (at 0.75 and 1.8 THz) and one minimum (at 1.25 THz). These represent constructive (*λ*/4 cavity conditions) and destructive (*λ*/2 cavity condition for the latter) interferences inside the cavity resonator. As the thickness decreases, these features shift toward higher frequencies with increasing magnitude in the first maximum as expected (*d* ≃ *λ*/4*n*). Figure 4 shows a summary of the improvements in peak-to-peak signal amplitude in the time domain (top) and magnitude of interference maxima position in the spectral domain (bottom). Continuous increase of the peak-to-peak amplitude is observed with decreasing thickness of the substrates with 1.2× increase in the time domain and 2× in the frequency domain for 25 µm cavity. From a frequency spectra point of view, it is possible to modify the emission bands of the STE by choosing the corresponding thickness of the substrate, with the enhancement in magnitude reaching double that of the STE without THz cavity.

To further investigate the influence of the cavity length on the frequency bandwidth of the STE, we performed several simulations by the finite element method (FEM) in commercially available software (COMSOL Multiphysics) and a custom frequency-domain nonstationary field dynamics code. The latter approach facilitates the simulation of time-dependent NIR excitation within STEs and the subsequent pulsed terahertz emission. This is accomplished through the application of the Scattering Matrix formalism (S-formalism) and Fourier series decomposition [25], [26], [27]. The method involves the coherent summation of the calculated field at each frequency component, resulting in a nonstationary/pulsed response and Poynting’s theorem for the time-evolution of the absorption, serving as the current source for dipole emission. The main advantage of S-formalism is its numerical stability, particularly in structures that combine thin metallic layers with thick multilayer structures. The results of simulated terahertz emission are corrected for the instrument function derived from measurements of a well-known reference sample (i.e., without cavity).

**Figure 5:**
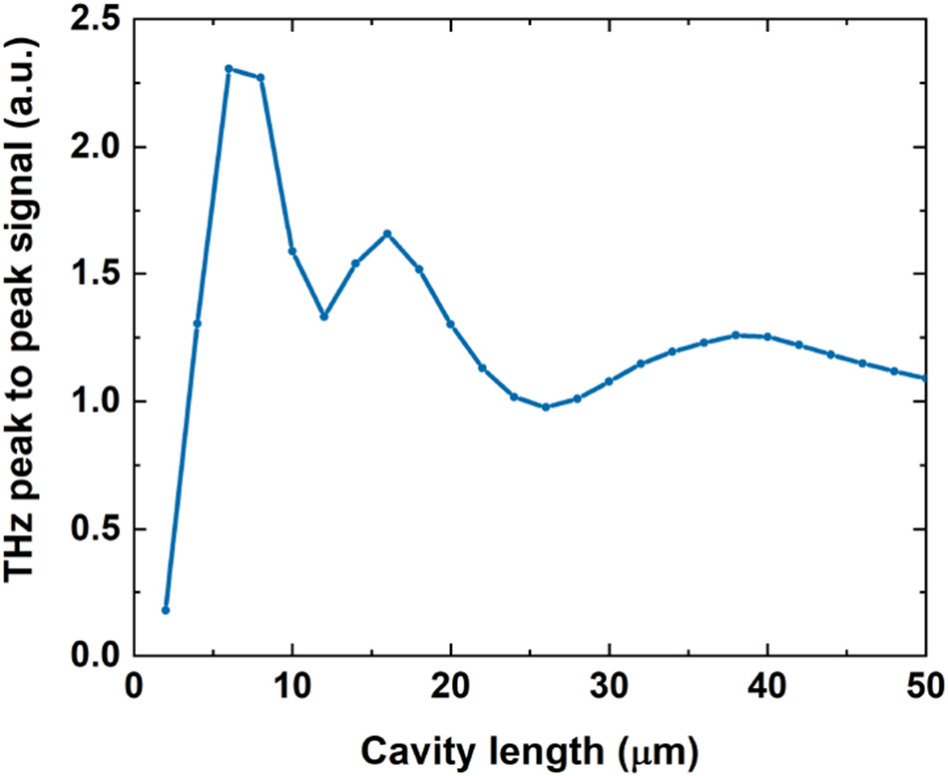
Simulated peak-to-peak THz signal by Scattering Matrix formalism showing maximum potential enhancement of the peak-to-peak field for thickness of 6 µm.

The electric field simulation results for the time domain (Figure 3(b)) and frequency domain (Figure 3(d)) show an excellent agreement with the measured data. The 39 µm cavity frequency components around 2 THz, however, overestimate the magnitude by a factor of 2, possibly a result of higher losses in type A sapphire. These simulations can also be used as a tool to investigate the optimal thickness to, for example, avoid the deconstructive interference in the measured spectral bandwidth. Figure 5 shows the peak-to-peak field in the time domain as a function of cavity thickness, predicting the highest field enhancement of 2.3 for a cavity with a thickness of 6 µm. Although this would be difficult to realize using sapphire materials, this would be relatively easily achievable using silicon-based membranes.

**Figure 6:**
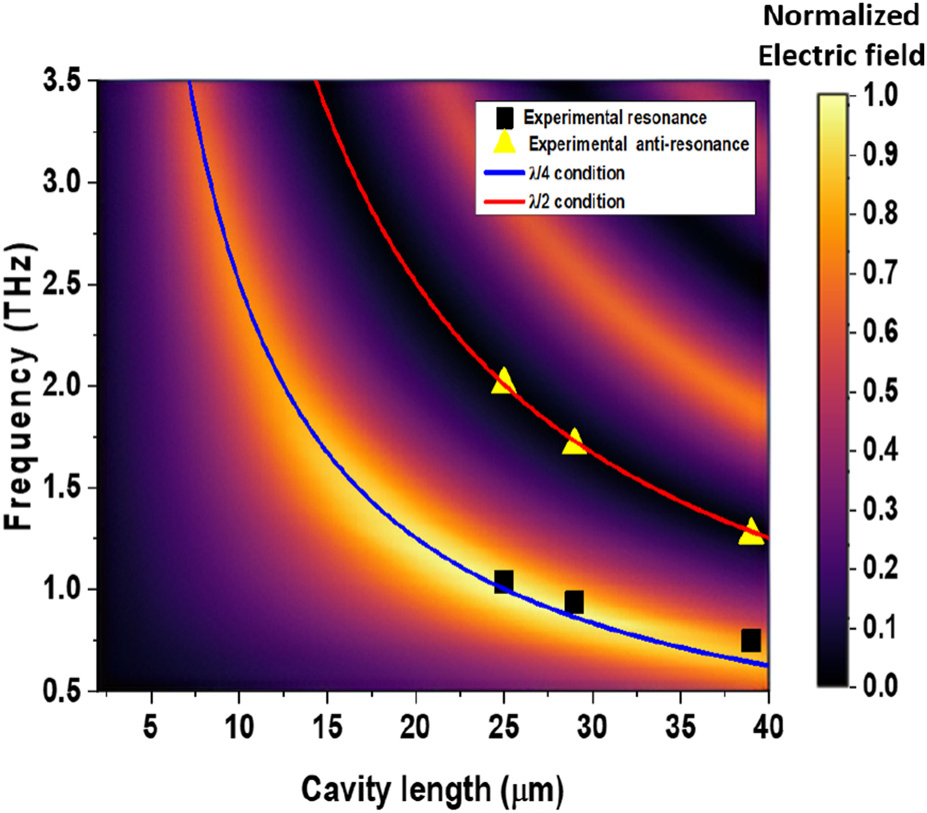
The FEM (COMSOL) simulated electric field as a function of frequency and cavity length (sapphire substrate thickness). The *λ*/4 and the *λ*/2 conditions are represented by a blue and a red line, with points showing experimental results for emission peak and emission gap.

**Figure 7:**
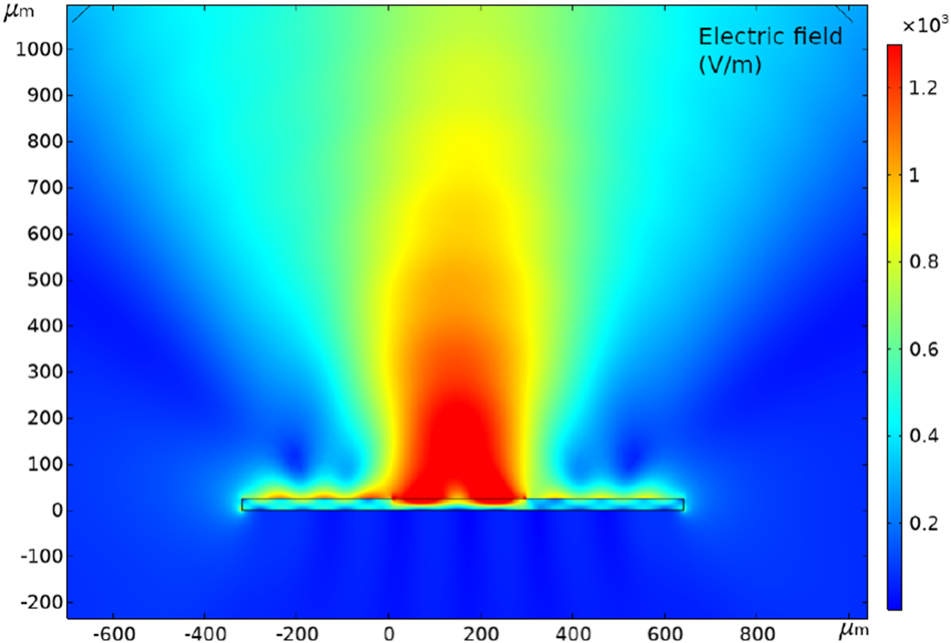
Example of electric field simulation by FEM (COMSOL) for 25 µm thick cavity at 1 THz resonance.

Regarding FEM simulations, Figure 6 shows the field collected in the far field from the emitter as a function of frequency and sapphire cavity thickness based on the simulations of the radiated field. This FEM approach is based on the terahertz emission being simulated with a transient current generated on the sample surface at the interface with air. Note that the optical pump generation mechanism is not included and, therefore, the second excitation owing to the transmission of part of the NIR pulse through the sapphire and its reflection back to the STE is not taken in account. Figure 7 shows an example of the simulated 2D radiated field at 1.0 THz emission, which is used to calculate the emitted field as a function of frequency of Figure 6. A resonant behavior is observed with a shift of the peak emission frequency toward higher THz values as the cavity thickness is reduced. The *λ*/4 and the *λ*/2 condition are represented by a blue line and a red line, respectively. The experimentally measured emission peak and emission gap of the 25 µm, 29 µm, and 39 µm cavity emitters are represented in the plot by squared black dots and triangular yellow dots, respectively. This plot shows that all three experimental gaps and peaks fit very well with the *λ*/4 condition and the *λ*/2 condition.

**Figure 8:**
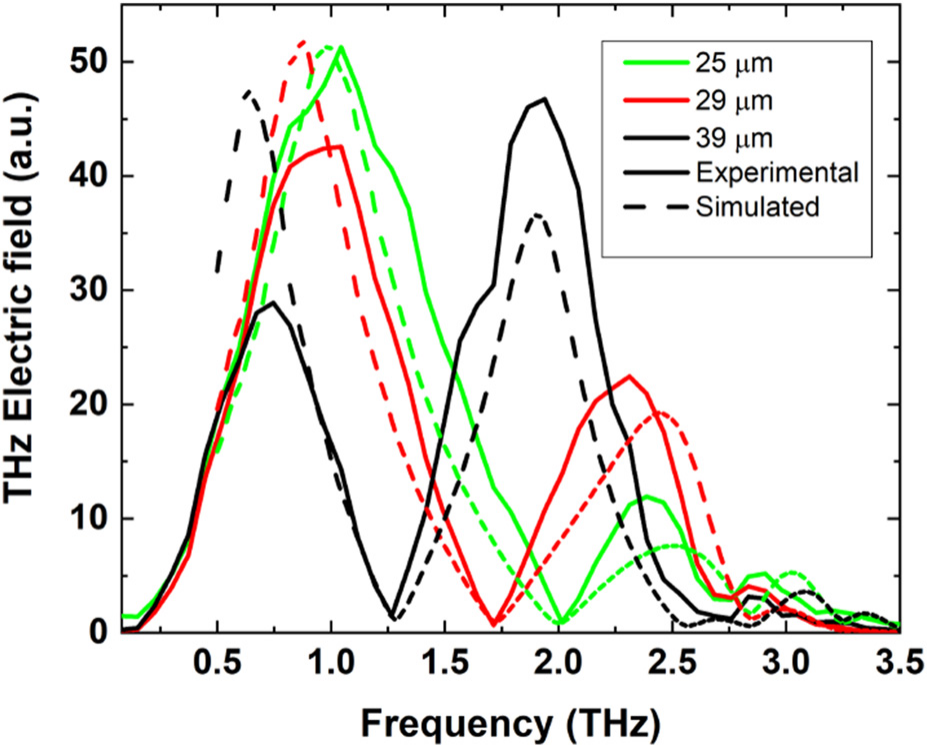
Comparison of experimental (continuous line) and spectra simulated by FEM (COMSOL, dashed line) as a function of frequency for the four different cavity thicknesses.

Figure 8 shows a comparison between experimental and FEM simulated spectra of the cavity STEs. Positions of maxima and minima fits well despite an overestimate of the first peak for the 29 and 39 µm cavities, which can be a result of the extra propagating NIR pulses owing to the optical transparency of sapphire (presence of total 4 THz pulses in experimental results – see above), while the FEM simulations include only the two THz pulses.

## 5 Toward THz polarization control

**Figure 9:**
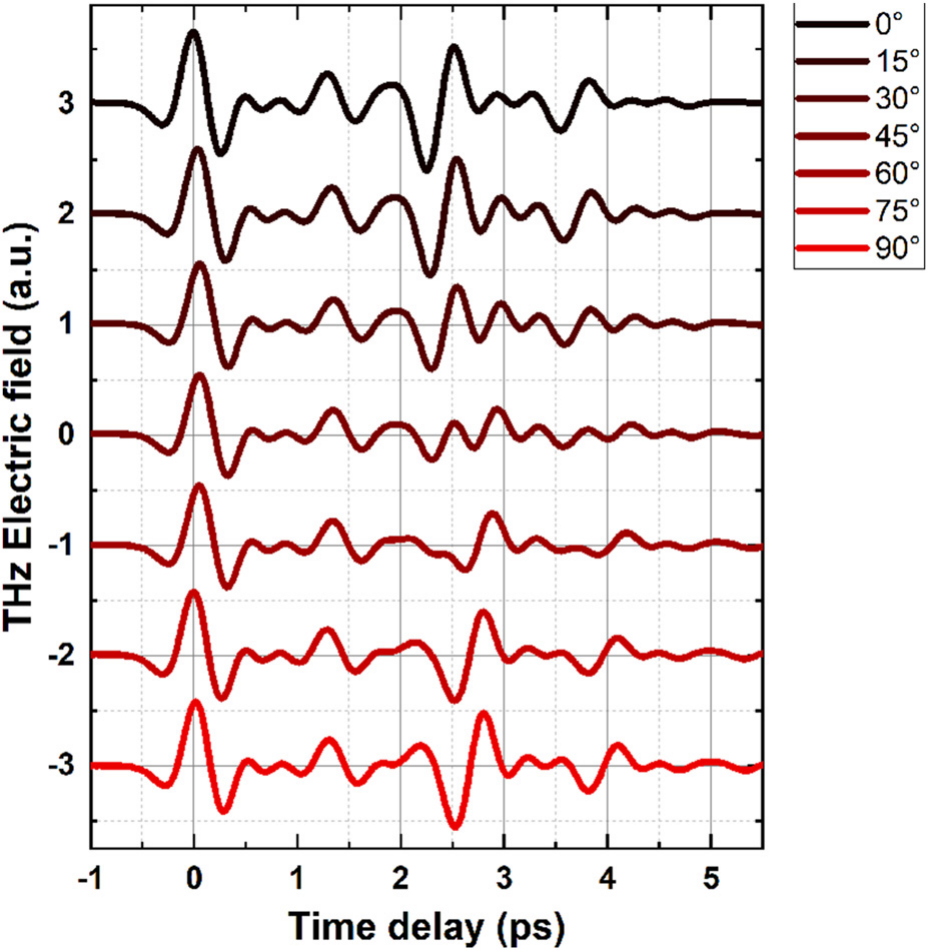
Change of THz time domain signal of STE sample on 111 µm thick substrate as a function of the rotation of the substrate crystal. Shift of time traces is a result of the birefringence in A-type sapphire. Only horizontal polarization is detected.

As a perspective of this work, we study the potential of integrated polarization control using the birefringent nature of the single crystal sapphire, where ordinary and extraordinary refractive indices at 1 THz are *n*_*o*_ = 3.07 and *n*_*e*_ = 3.41, respectively [21]. The sapphire substrates used in our samples are from A-cut sapphire, in which *n*_*o*_ and *n*_*e*_ are perpendicular to each other in the plane of the sample. Therefore, by rotating the sample, refractive index for THz pulse changes as the substrate behaves like waveplate. This can be used to change the polarization of the reflected THz pulse. Figure 9 shows change in horizontal polarization of emitted THz pulse during rotation of the 111 µm thick substrate crystal from fast (*n*_*o*_) to slow (*n*_*e*_) axis.

It can be seen that the time domain signal for the two last pulses (reflections from the back mirror) changes with the angle of rotation. Signals for 0° and 90° have the same shape but are shifted in time owning to higher optical path length for pulses traveling along the slow axis with higher refractive index *n*_*e*_.

**Figure 10:**
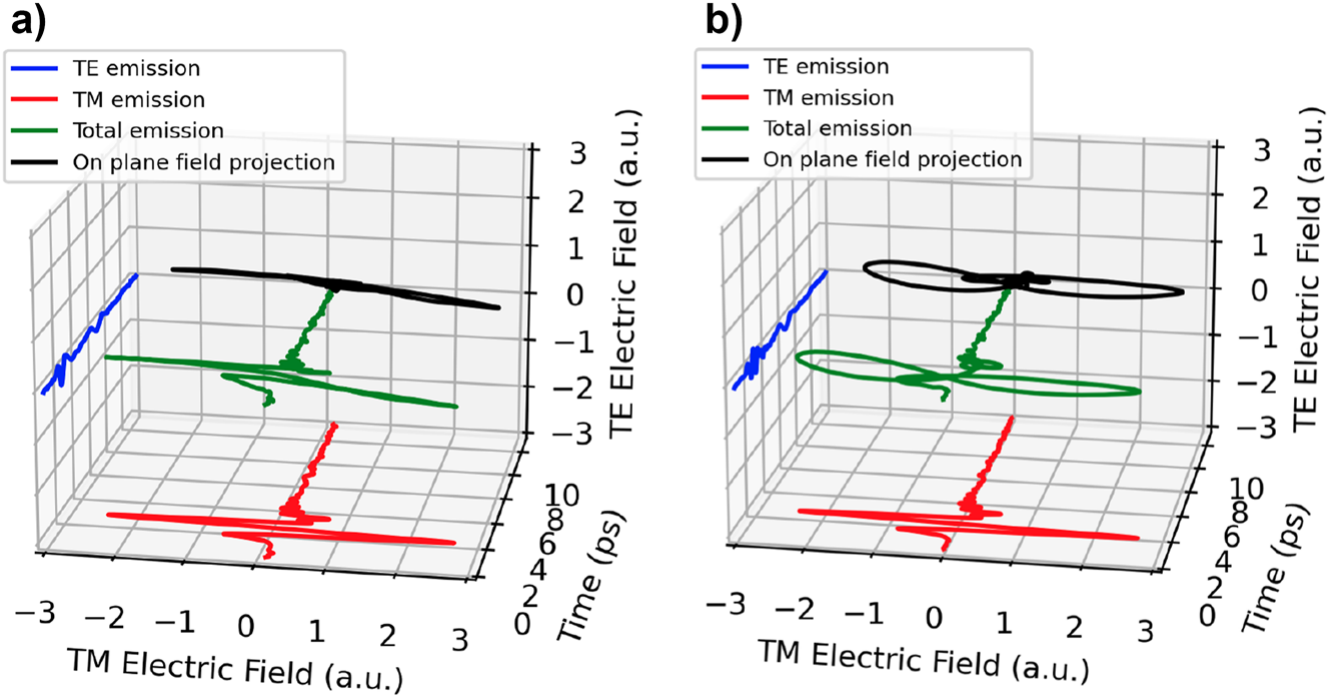
Transverse electric (red) and transverse magnetic (blue) experimental fields of the 25 µm thick cavity for azimuthal angle with (a) the smallest TE component (0°) and (b) with the highest TE component (45°). The green curve is the total emission to highlight the elliptical nature of the emitted pulse and the black curve is the 2D projection on the back plane.

The situation is different for the thinnest cavity sample, where the change of polarization is not so high owning to the short path in the birefringent substrate. Figure 10 shows time domain signal from the 25 µm cavity for orientation 0° and 45° and where the horizontal and vertical THz electric field polarizations are measured. The former contains virtually only horizontally polarized THz pulses, but at 45°, the ellipticity of the THz pulse is noticeable. This ellipticity is created by the two THz pulses where the first is polarized horizontally and the second can have arbitrary polarization (depending on the thickness and rotation angle of the substrate). Note that here the substrate acts as monochromatic waveplate and the ellipticity of the second pulse varies for frequency components so would be on most interest for narrowband THz generation [28]. By designing the substrate not only for THz cavity but also for proper phase retardation over larger bandwidth of the THz pulses (i.e., achromatic waveplate), an STE source that can produce any desired linear, circular, or any arbitrary elliptical polarization can be potentially realized. This would be of particular interest for THz time domain ellipsometry.

## 6 Conclusions

The aim of our presented study was to show the incorporation of STEs with THz cavities, permitting to enhance and control the spectral field of the emitted THz pulse. All previous approaches have been based on enhancing the optical absorption using optical cavities with no photonic control of the THz emitted pulse. Here, this was achieved by incorporating a back metallic reflector on thin transparent sapphire substrates, effectively creating quarter wavelength THz cavities. Several STE samples on sapphire substrates were investigated with thickness 111 µm, 39 µm, 29 µm, and 25 µm, with a deposited back metallic mirror. THz emission time domain spectroscopy in reflection configuration revealed and improved maximum peak amplitudes of the THz signal in both time domain and frequency domain with the thinnest cavity showing the most important enhancements (twice the spectral amplitude at maximum emission), with single cycle emission. The frequency domain showed interference effects with maxima and minima in the spectra that shifted toward higher frequencies with decreasing thicknesses. Experimental results were compared with simulations by finite element method and a custom frequency-domain nonstationary field dynamics approach with a very good agreement. While our design is currently applicable only in reflection configuration, it would be possible to realize an emitter working in transmission configuration using a material with a cavity that is transparent for NIR pump and reflecting for the generated THz beam. These proof-of-principle of STE-based THz cavities can be further improved, where the developed simulations tools predicting improvements of doubling the temporal field for thinner cavities. This approach is scalable and could also be easily combined with optical dielectric mirrors or photonic cavities to further enhance the efficiency or the maximum THz emitted fields from STEs. As well as improving the STEs, this work of THz field enhancements could also open up perspectives of efficient even-order harmonic generation and THz down-conversion from spintronic heterojunctions [29].
